# Bacterial Histidine Kinase and the Development of Its Inhibitors in the 21st Century

**DOI:** 10.3390/antibiotics13070576

**Published:** 2024-06-22

**Authors:** Ragib Ahsan, Sumaiya Kifayat, Krishan Kumar Pooniya, Sunita Kularia, Bhavani Sailu Adimalla, Bharat Kumar Reddy Sanapalli, Vidyasrilekha Sanapalli, Dilep Kumar Sigalapalli

**Affiliations:** 1 Department of Pharmacy, NIMS Institute of Pharmacy, NIMS University, Jaipur 303121, Rajasthan, Indiakifayatsumaiya@gmail.com (S.K.);; 2 Department of Pharmacology, NIMS Institute of Pharmacy, NIMS University, Jaipur 303121, Rajasthan, India; kulariasunita01@gmail.com; 3 Department of Pharmaceutical Analysis, Vignan Pharmacy College, Jawaharlal Nehru Technological University, Vadlamudi, Guntur 522213, Andhra Pradesh, India; bhavanisailu18@gmail.com; 4 Department of Pharmacology, School of Pharmacy & Technology Management, SVKM’s Narsee Monjee Institute of Management Studies (NMIMS) Deemed to-be-University, Jadcherla 509301, Hyderabad, India; bharathsanapalli@yahoo.in; 5 Department of Pharmaceutical Chemistry, School of Pharmacy & Technology Management, SVKM’s Narsee Monjee Institute of Management Studies (NMIMS) Deemed to-be-University, Jadcherla 509301, Hyderabad, India; 6 Department of Biochemistry, University of Washington, Seattle, WA 98195, USA

**Keywords:** bacterial histidine kinase, two-component signaling system, antibacterial resistance, bacterial histidine kinase inhibitors

## Abstract

Bacterial histidine kinase (BHK) is a constituent of the two-component signaling (TCS) pathway, which is responsible for the regulation of a number of processes connected to bacterial pathogenicity, virulence, biofilm development, antibiotic resistance, and bacterial persistence. As BHK regulation is diverse, inhibitors can be developed, such as antibiotic synergists, bacteriostatic/bactericidal agents, virulence inhibitors, and biofilm inhibitors. Inhibition of essential BHK has always been an amenable strategy due to the conserved binding sites of the domains across bacterial species and growth dependence. Hence, an inhibitor of BHK might block multiple TCS regulatory networks. This review describes the TCS system and the role of BHK in bacterial virulence and discusses the available inhibitors of BHK, which is a specific response regulator with essential structural features.

## 1. Introduction

Bacterial infections have a substantial effect on global health. However, the discovery of wonder medications known as “antibiotics” offered consistent health advantages, reduced infections and decreased patient mortality during the last decade [1]. Moreover, the extensive use and misuse of antibiotics exacerbates selective pressure on microbes, leading to antimicrobial resistance (AMR). The global scope of the problem, as well as the impact of AMR on human health, health-care expenses, and society, remains largely unclear [2]. AMR is a complicated worldwide public health concern, and no single or simple solution will suffice to fully control the emergence and spread of pathogenic organisms resistant to existing antibacterial medications [3]. AMR is caused by a number of different mechanisms, such as drug or target inactivation (penicillinases, cephalosporinases, carbapenemases, and β-lactamases), binding site modifications (PBP2a in *Staphylococcus aureus*, which changes the cross-linking target of the peptidoglycan layer in *Enterococcus faecium* and *Enterococcus faecalis*), and the development of resistance to AMR (the reduced level of OprD porin protein in *Pseudomonas aeruginosa* exhibits resistance against imipenem) [4,5,6,7]. In 2010, *Neisseria*, *Staphylococcus*, and *Enterobacteriaceae* developed resistance to the antibiotic ceftaroline (2010); *Staphylococcus* developed resistance to both linezolid (2000) and daptomycin (2003) in 2001; and *Acinetobacter* and *Pseudomonas* developed resistance to these antibiotics in 2004 and 2005, respectively. According to statistics from the WHO, 1.27 million patients died due to infections caused by resistant bacteria in 2019 [8,9,10]. Furthermore, the mortality rate might increase to 10 million patients each year by 2050, as reported by O’Neill. Therefore, immediate action is needed to counteract the emergence and rampant dissemination of AMR [11].

Many targets have been investigated for the development of antibacterial drugs [1]. The cell wall biosynthesis process has been widely investigated and validated as an antibacterial target of the β-lactam and glycopeptide classes of antibiotics [12]. The fatty acid production pathway has also been validated by the widespread use of well-known medications such as isoniazid, an antitubercular treatment, and triclosan, an antiseptic [13]. Bacterial folate biosynthesis is a well-known and appealing target that involves many types of enzymes. However, the well-known targets are DHFR (dihydrofolate reductase) and DHPS (dihydropteroate synthase). DHFR has been verified by the use of drugs such as trimethoprim (an antifolate antibiotic) and pyrimethamine (an antiprotozoal agent). DHPS is another target that has been proven to be crucial for folate synthesis and was validated as a sulfonamide [14].

Another potent antibacterial target is the dual inactivation of DNA GyrB and ParE. By inhibiting these topoisomerases, DNA replication, repair, and catenation are prevented [15]. Furthermore, protein synthesis, primarily carried out by the molecular machinery known as ribosomes and translational machinery, is regarded as a vulnerable target for antibiotics [16]. Tetracyclines are widely used antibacterial medications that target protein synthesis (blocking the A site of the 30S subunit of the ribosome, thereby preventing the binding of aminoacyl t-RNA) [17]. Aminoglycosides interfere with the formation of initiation complexes of the 30S subunit [18]. During the transpeptidation cycle, macrolides interfere with the elongation of peptides [19]. Despite the fact that these antibiotics and their targets have been shown to be clinically significant, the increase in resistance necessitates the development of novel strategies. Several strategies already reported in the literature include structural modification, bacteriophage therapy, and targeting of the explored pathways with novel molecules.

None of these strategies were found to be effective in preventing AMR. Therefore, research is more focused on the discovery of novel untapped or unexplored pathways. One such attractive target is bacterial histidine kinases (BHKs). BHKs are constituents of bacterial two-component systems (TCSs), which are involved in primary signal transduction pathways. BHK is highly conserved among all bacterial species and has broad-spectrum activity. In addition, no human homologs or proteins with similar structures (with the exception that mammalian kinases possess comparable protein folds in the ATP domain) exhibit selectivity toward bacterial species [20]. Furthermore, BHK is important for bacterial survival, the inhibition or inactivation of which results in bacterial death. All these characteristics make BHK a potential antibacterial target. In this review, we discussed the biological significance of TCS-BHKs in the identification of new antibacterial agents as well as existing TCS-BHK inhibitors, which can be used further to develop new and diverse antibacterial agents.

## 2. TCS Signaling Pathway and BHK

**Figure 1:**
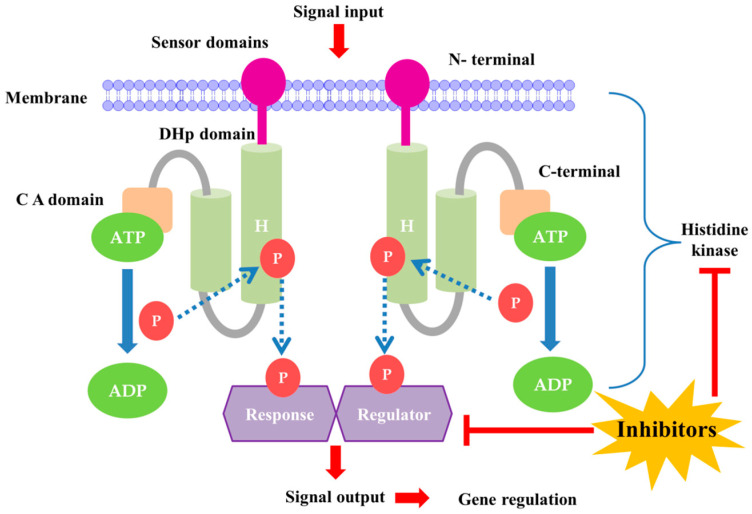
The TCS signaling pathway and role of bacterial histidine kinase in gene regulation. **DHp domain-**Dimerization and histidine phosphotransfer domain; **C A domain-**Catalytic and ATP binding domain; **H-box-**Highly conserved histidine residue; **P-**Phosphoryl group.

TCSs are considered appealing antibacterial targets because they are conserved in almost all bacterial species. In addition, the TCS is and involved in the regulation of a number of processes connected to bacterial pathogenicity, virulence, biofilm development, antibiotic resistance, and bacterial persistence. Although crucial for bacterial adaptability and fitness, only a few of these TCSs are considered essential for bacterial cell survival. However, some TCSs are not essential for bacterial survival in laboratory environments, but they enhance bacterial fitness by enabling adaptation to environmental changes. Certain responses are expressed by pathogenic bacteria in response to their host environment, and these responses typically rely on the TCS system. Another important consideration is the difference between targeting essential and nonessential TCSs in pathogens. Essential TCSs are essential for the survival and growth of bacteria. While inhibiting TCSs can efficiently kill or suppress bacteria, it can also lead to the swift development of resistance, as bacteria are under strong selective pressure to survive. On the other hand, nonessential TCSs often regulate virulence factors rather than basic survival. By targeting these systems, we can reduce the pathogen’s ability to cause disease without necessarily killing it, which may result in slower resistance development. For example, studies have shown that targeting the Agr system in *Staphylococcus aureus*, which is not essential for survival but crucial for virulence, can significantly diminish its ability to cause infections without inducing rapid resistance [21,22]. This approach could offer a more sustainable way to manage bacterial infections. TCS signaling involves autophosphorylation of a membrane-bound BHK, phosphotransfer of the phosphoryl group to a cognate response regulator (RR), and ultimately modulation of the expression of target genes (Figure 1) [23]. BHKs are present in both essential and nonessential TCSs. Appropriate phosphorylation levels of RR are tightly regulated by the phosphatase activity of BHK, RR, or a partner protein [23,24]. BHK autophosphorylation is mediated via the catalytic and ATP-binding (CA) domain, which binds ATP and phosphorylates BHK at a conserved histidine residue in the dimerization and histidine phosphotransfer (DHp) domain. The CA and DHp domains are conserved and present in all HKs, whereas the remaining sensor domains (periplasmic, PAS, GAF, HAMP) are variable and not present in all HKs [23].

The BHK CA domain is a desirable target for structure-based virtual screening and phenotypic screening of pharmacological inhibitors due to its conserved properties and crucial function in TCS signal transduction. The high level of sequence conservation in the CA catalytic site further suggests that inhibitors directed against this region will have broad-spectrum antibacterial effects. The CA domain is thus a promising BHK target location for the discovery and development of broad-spectrum antibiotics. Drug polypharmacology, which involves simultaneous inhibition of many targets, has been suggested as a method to prevent the emergence of drug resistance to novel antibiotics [25,26,27,28]. Because bacteria have several TCSs, inhibitors of the highly conserved CA domain are likely to shut down a number of signaling pathways, impairing the bacteria’s capacity to quickly adapt to environmental changes, including those that occur during an infection of the host. TCS inhibition may not be bactericidal for some bacteria, but it is likely to limit efficient growth, lowering survival capacity [23] (Figure 1). The ATP-binding Bergerat fold found in the CA domain of many human protein families, which is also present in essential proteins such as Hsp90, is one potential drawback of BHKs. The Bergerat fold may cause BHK autophosphorylation inhibitors (HKAIs) to have off-target effects on human ATP-binding domains and may also cause toxicity to mammalian cells. This fold is present in both microbial and human ATP-binding protein domains [29].

### TCS-BHK Inhibitors

For almost 20 years, TCSs have been identified as viable antibacterial therapeutic targets. Some TCSs are essential or required for bacterial growth. Furthermore, given the high degree of conservation among TCS active sites and the occurrence of several TCSs in every bacterium, an inhibitor with broad-spectrum activity that targets various TCS regulatory networks should be identified. Overall, targeting TCSs is likely to effectively disable bacteria’s ability to adapt to environmental and physiological changes. The availability of crystal structures of BHK has made the design of BHK inhibitors possible. In the current review, we discussed novel BHK inhibitors with different response regulators identified in the literature (Table 1).

**Figure 2:**
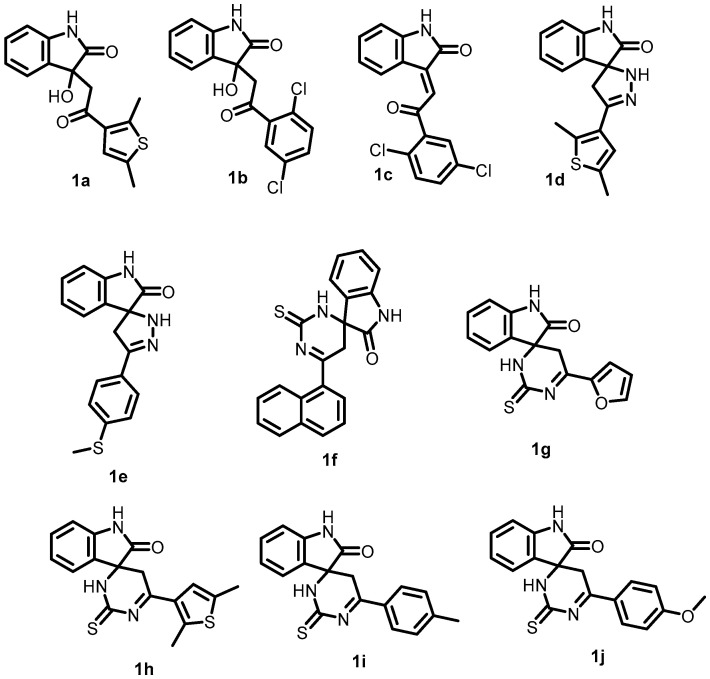
Chemical structures of isatin derivatives (**1a**–**1j**).

In 2022, Radwan et al. synthesized a series of novel isatin derivatives with either β-hydroxyketone or chalcone moieties and examined their antibacterial activity. These compounds (**1a**–**1j**) (Figure 2) exhibited potent activity against *S. aureus* in the range of 0.044–0.057 mmol/L (MIC). Among these compounds, **1a** showed the most potent antibacterial activity, with an MIC of 0.026 mmol/L. The activity of **1a** against *S. aureus* was explained by its significant docking score values (glide score −36.231 kcal mol^−1^, electrostatic energy −0.697 kcal mol^−1^, and van der Waals energy −35.534 kcal mol^−1^) within the binding site of BHK (*S. aureus*) (PDB: 5C93). Compound 1a could be further optimized for the development and synthesis of more potent antibacterial agents [48].

**Figure 3:**
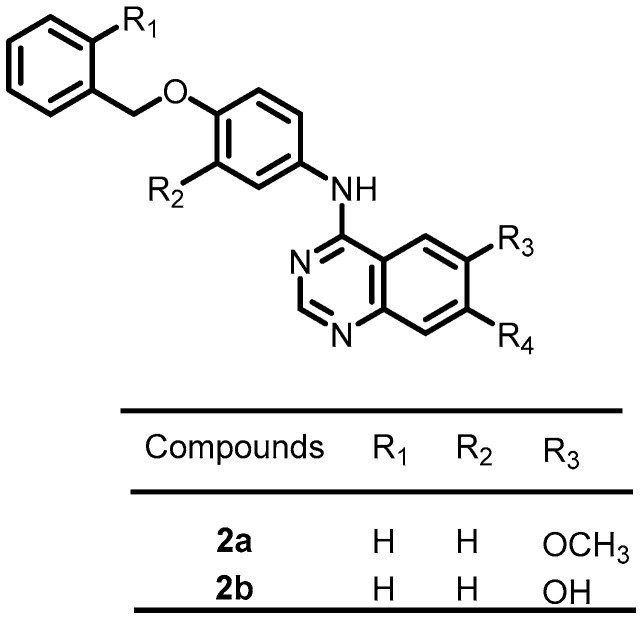
Chemical structures of kinase inhibitors **2a**–**2b**.

Focusing on the discovery of novel antibacterial agents in 2020, Carabajal et al. screened 686 compounds from the published kinase inhibitor set (PKIS), a compound library published by GlaxoSmithKline, to identify inhibitors of PhoP/PhoQ in *S. typhimurium*. The results demonstrated that a series of compounds with quinazoline scaffolds exhibited potent and selective downregulation of PhoP/PhoQ-activated genes. Among these quinazoline derivatives, **2a** and **2b** (Figure 3) showed more potent antibacterial activity, with IC_50_ values of 6.9 and 3.2 µM, respectively. Furthermore, these compounds can emerge as appealing lead molecules for the development of antibacterial agents [49].

**Figure 4:**
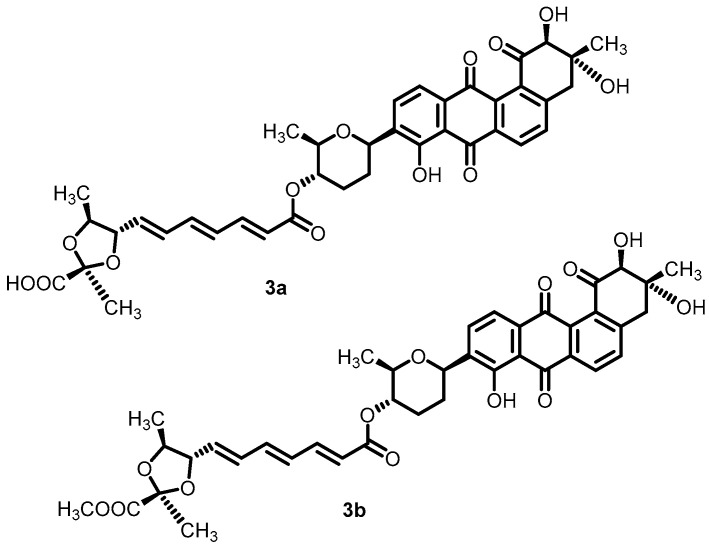
Chemical structures of waldiomycin (**3a**) and its methylester derivative (**3b**).

In an effort to discover novel antibacterial agents, waldiomycin (**3a**) and its methyl ester derivative (**3b**) (Figure 4) were identified as novel BHK inhibitors. Waldiomycin, a methyl ester derivative, exhibited significant inhibitory activity against the Walk-type H-box region, with IC_50_ values of 10.2 and 75.8 µM, respectively. The results demonstrated that the binding interactions of ligands with WalK-BHK could be studied further for the development of novel antibacterial agents [50].

**Figure 5:**
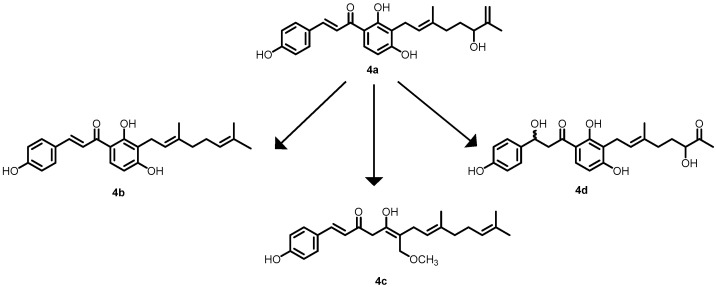
Chemical structures of the xanthoangenol derivatives (**4a**–**4d**).

In another study by Mizar et al. in 2018, xanthoangenol B 1 (**4a**) was identified using a GFP (green fluorescent protein) reporter system that was previously used to identify SaeRS TCS (response regulator in *S. aureus*) inhibitors obtained from plants. Approximately four derivatives (xanthoangenol (**4b**), xanthoangenol (**4c**) and PM-56 (**4d**)) (Figure 5) were identified and screened for their antibacterial activity. Among them, **4a** and **4d** demonstrated excellent inhibitory activity against SaeRS, with IC_50_ values of 2.1 and 4.3 µM, respectively [51].

**Figure 6:**
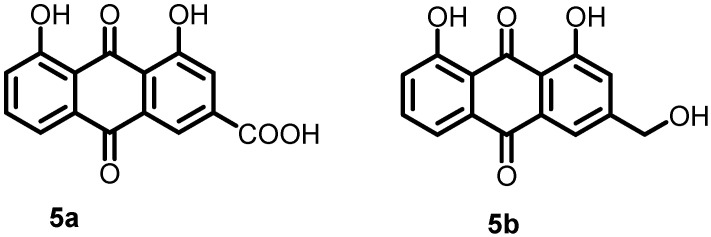
Chemical structures of the traditional Chinese medicine monomers rhein (**5a**) and aloe emodin (**5b**).

In 2019, Zhang et al. developed a system based on artificial proteoliposomes and used it for screening AgrC inhibitors. A library of traditional Chinese medicine (TCM) monomers was selected and screened for ArgC inhibitory activity. The results showed that the two TCM monomers rhein (**5a**) and aloe emodin (**5b**) (Figure 6) inhibited AgrC autophosphorylation with IC_50_ values of 13.7 and 62.2 μM, respectively. Furthermore, these compounds inhibited the growth of *S. aureus* in a dose-dependent manner, with MIC values of 32 and 64 μg/mL, respectively [52].

**Figure 7:**
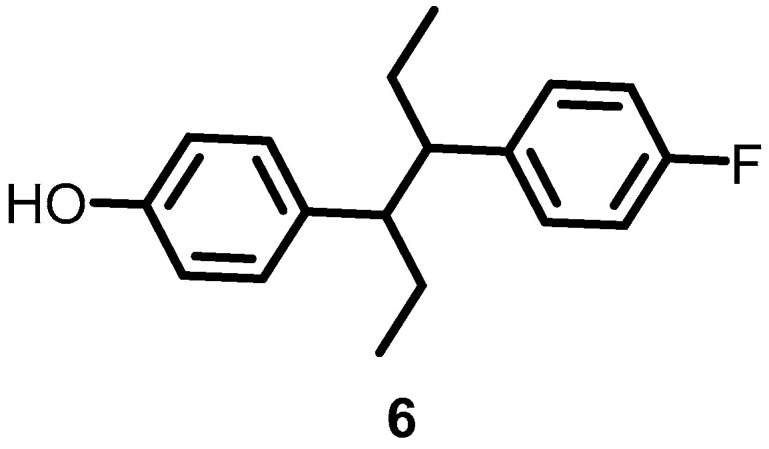
Chemical structures of fragment **6**.

In 2016, Velikova et al. reported the identification of putative BHK autophosphorylation inhibitors by combining in silico and in vitro fragment-based screening. Among the screened fragments, compound **6** (Figure 7) was the most potent compound, inhibiting the autophosphorylation of BHK in a concentration-dependent manner, with IC_50_s against *S. aureus* and *E. coli* BHK PhoR of 212 and 16 μM, respectively [31].

**Figure 8:**
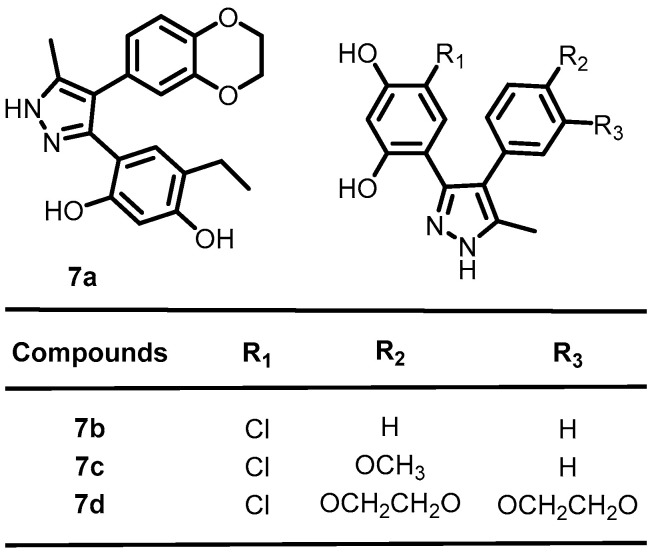
Chemical structures of diaryl pyrazole-based derivatives **7a**–**7d**.

Continued efforts to increase the potency of molecules against BHK led to the discovery of novel heterocycles. In 2017, Vo et al. demonstrated that repurposing diaryl pyrazole-based ATP-competitive (HSP90) inhibitors as effective antibacterial agents targeting BHKs is a promising strategy for the development of newer antibiotics. A total of nine CCT018159 (**7a**, Figure 8) derivatives were synthesized and evaluated against multiple BHKs (PhoQ, DivJ, and Cck). Compounds **7b**, **7c**, and **7d** (Figure 8) showed favorable properties, both for the inhibition of CckA (*C. crescentus*) and PhoQ (*Salmonella*), which are essential for virulence. The results confirmed that the presence of a chlororesorcinol ring was essential for potent activity within the series. In summary, this study identified a pathway for the development of HSP90 inhibitors as novel antibacterial agents [30].

**Figure 9:**
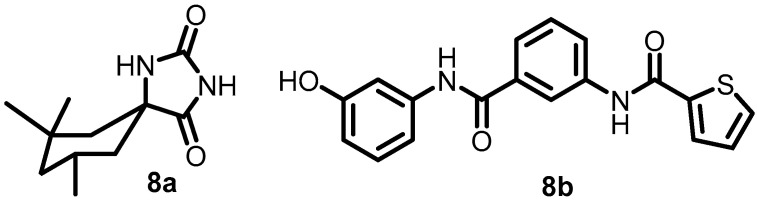
Chemical structures of DosRST inhibitors **8a**–**8b**.

In 2017, Zheng et al. used whole-cell phenotypic high-throughput screening to screen a small-molecule library of approximately 540,000 compounds to identify new DosRST inhibitors. Compounds **8a** and **8b** (Figure 9) were identified as potential antibacterial agents. Compound **8a** reduced the autophosphorylation of DosS with an IC_50_ of 1.9 µM, and **8b** inhibited the autophosphorylation of both DosS and DosT with IC_50_s of 0.5 and 5 µM, respectively [53].

**Figure 10:**
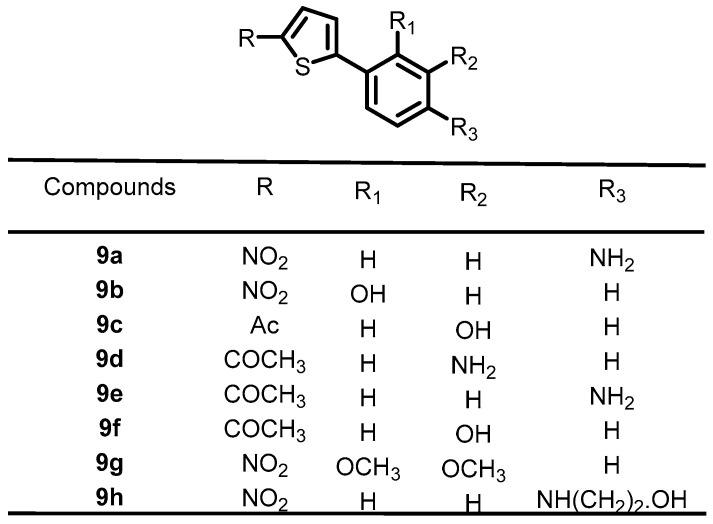
Chemical structures of thiophene derivatives **9a**–**9h**.

In 2016, Boibessot et al. synthesized a series of thiophene derivatives and screened them for their antibacterial activity. Among them, eight compounds (**9a**–**9h**) (Figure 10) were found to inhibit the autophosphorylation activity of the BHKs WalK, PhoR, and ResE from *B. subtilis*, with IC_50_ values ranging from 52.81–196.9, 1.63–122.6, and 20.3–243.9 μM, respectively. These lead compounds can be used as a starting point for the development of novel antibacterial agents [36].

**Figure 11:**
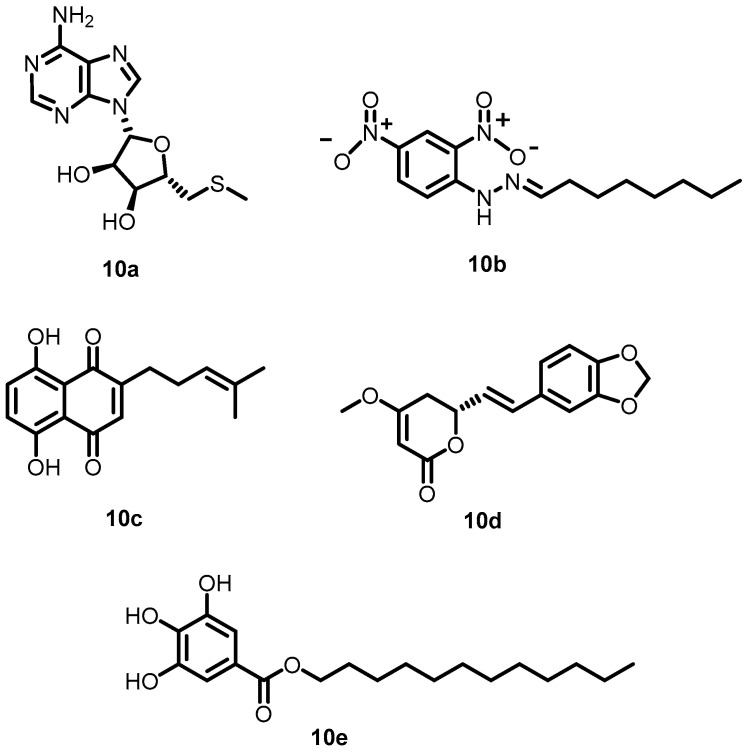
Chemical structures of TCM derivatives **10a**–**10e**.

Prompted by TCM monomer activity against BHK, Zhang et al. in 2015 explored the other TCM monomers **10a**–**10e** (Figure 11) using structure-based virtual screening of a natural TCM monomer library. These compounds specifically inhibited the autophosphorylation of VicK in a dose-dependent manner, with IC_50_ values of 3.8, 5.4, 15.4, 4.6, and 9.1 µM, respectively. In addition, the compounds exhibited potent antibacterial activity (**10a**: 37.1 µg/mL; **10b**: 38.5 µg/mL; **10c**: 17 µg/mL; **10d**: 68.5 µg/mL; **10e**: 21 µg/mL) against *S. pneumoniae* [54].

**Figure 12:**
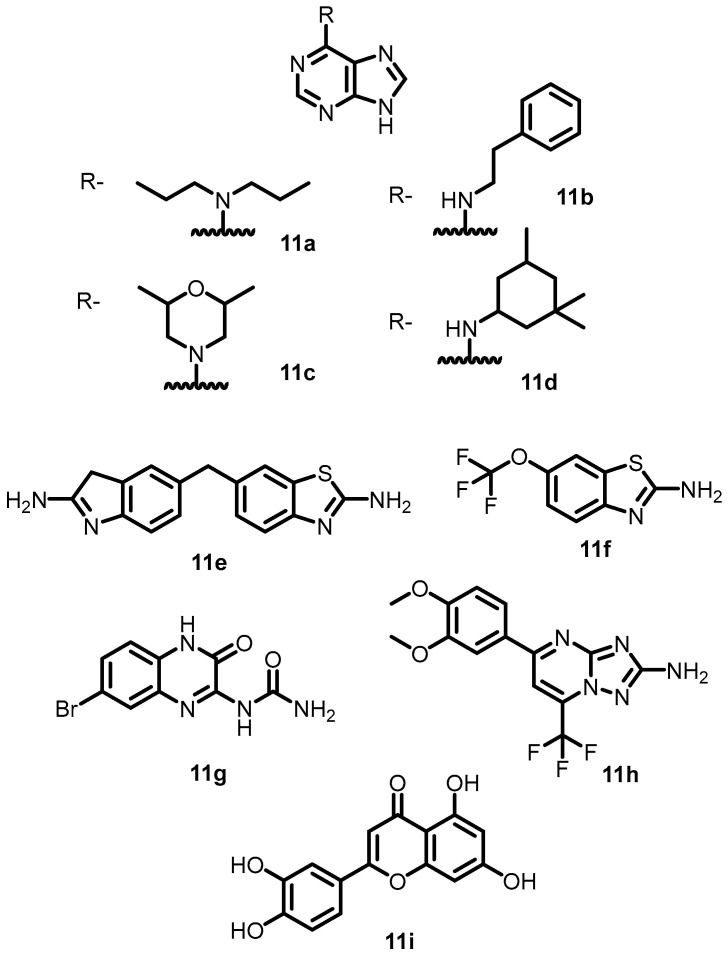
Chemical structures of adenine derivatives **11a**–**11i**.

In 2015, Wilke et al. elucidated the active site of BHK using an HTS-FP displacement assay. The results demonstrated that nine compounds exhibited potential inhibitory activity against different BHKs. Among them, four compounds (**11a**–**11d**) (Figure 12) containing adenine moieties possess significant targetable inhibitor space within the binding pocket. The other five compounds (**11e**–**11i**) (Figure 12) that possess unique chemical structures were found to be more potent, as evidenced by their IC_50_ values. These compounds could be utilized for the production of multitargeted, TCS-mediated antibiotics with innovative modes of action [46].

**Figure 13:**
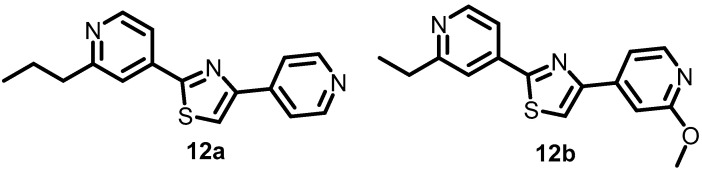
Chemical structures of diarylthiazole derivatives **12a**–**12b**.

In 2014, Bellale et al. discovered a particular class of diarylthiazole compounds (Figure 13) that had potent inhibitory activity against PrrBA TCA, which is required for the viability of *M. tuberculosis*. Over 40 diarylthiazole derivatives, such as **12a** and **12b**, which demonstrated remarkable antibacterial activities with MICs of 0.4 and 0.25 µg/mL, respectively, were subsequently developed, and the majority of these derivatives exhibited favorable physicochemical characteristics and significant MICs against *M. tuberculosis* (MIC ≤1 µg/mL) [55].

**Figure 14:**
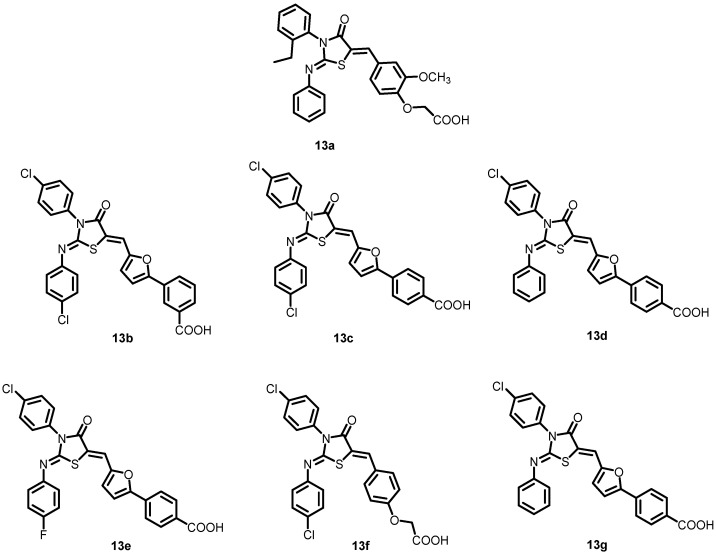
Chemical structures of compounds **13a**–**13g**.

In another study by Liu et al. in 2014, six analogs of thiazolidine (**13a**) (**13b**–**13g**) (Figure 14) were developed and created by altering functional groups to enhance the antibacterial activity and decrease the toxicity of **13a**. The results indicated the inhibitory effects of these compounds on the autophosphorylation of WalK, with IC_50_ values ranging from 24.2 to 71.2 µM. With MICs ranging from 1.5 to 6.3 µM, these compounds exhibited strong antibacterial activity against *S. epidermidis* and *S. aureus*, including clinical methicillin-resistant *S. epidermis* (MRSE) and MRSA, which were dramatically improved compared to **13a** [35].

**Figure 15:**
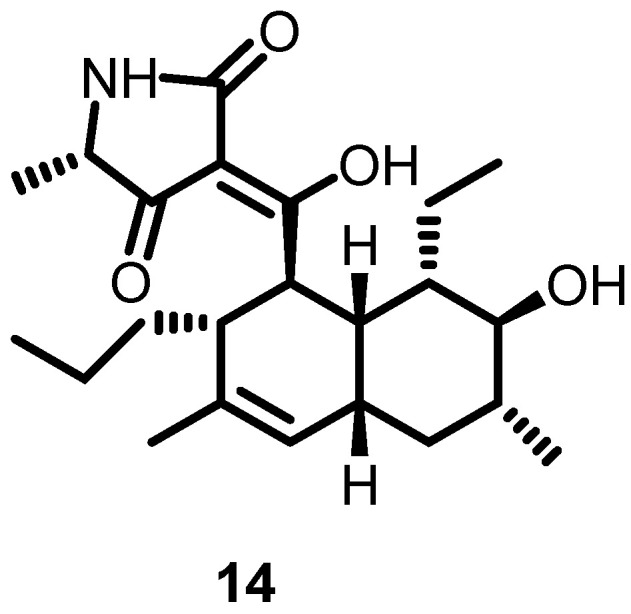
Chemical structure of signermycin B (**14**).

In 2012, Watanabe et al. screened more than 10,000 Streptomyces extracts by using differential growth assays and identified signermycin B (**14**) (Figure 15) as a potent compound that interfered with the WalK dimerization domain. Furthermore, its inhibitory activity against WalK was evaluated for different bacterial species (*S. aureus*, *E. faecalis*, *B. subtilis*, and *S. mutans*), and IC_50_ values ranging from 37–62 µM were calculated. These results demonstrated that the WalK dimerization domain could serve as a potent binding site, and further optimization of singermycin B could lead to the development of novel antibacterial agents [40].

**Figure 16:**
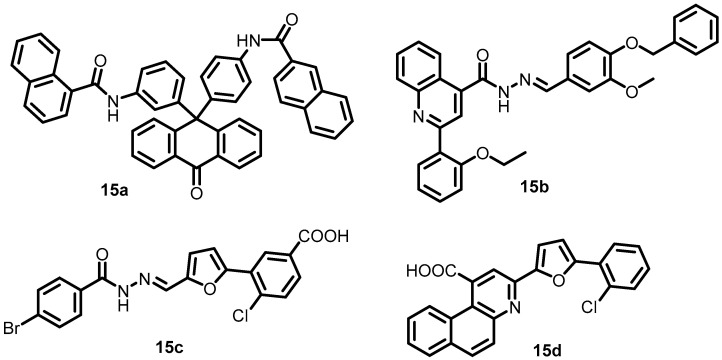
Chemical structures of PhoQ inhibitors **15a**–**15d**.

In another study, Cai et al. in 2011, identified four compounds, **15a**–**15d** (Figure 16), as possible PhoQ inhibitors using HTS and enzymatic activity-coupled assays. These four compounds had significant binding affinities to the *S. flexneri* PhoQc protein in the surface plasmon resonance (SPR) response and inhibited the autophosphorylation activity of *S. flexneri* PhoQc (KD = 4.50, 10.6, 7.56, and 9.40 µM, respectively). The IC_50_ values of these four compounds calculated during the luminescent kinase assay were 69.37 (**15a**), 48.9 (**15b**), 7.99 (**15c**), and 27.2 (**15d**) μM. The results showed that all four putative PhoQ inhibitors were able to reduce Shigella virulence [56].

**Figure 17:**
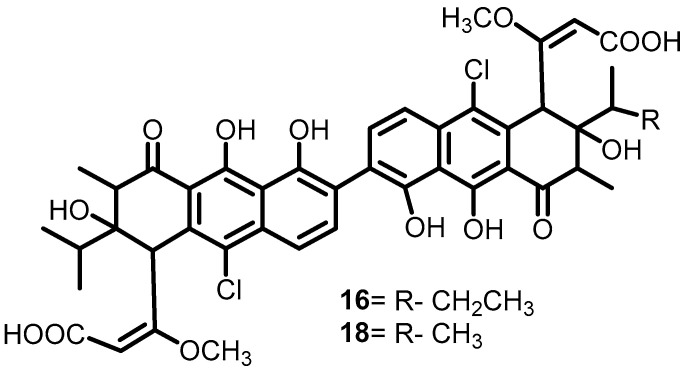
Chemical structure of walkmycin C (**16**).

Another study by Eguchi et al. in 2011 investigated the effect of walkmycin C (**16**) (Figure 17) on WalK BHK in *B. subtilis* and *S. aureus*. Furthermore, walkmycin also exhibited significant activity against the cytoplasmic domains of VicK (IC50: 2.53 μg/mL), CiaH (IC50: 4.29 μg/mL), and LiaS (IC_50_: 4.96 μg/mL) of *Streptococcus mutans*. Moreover, it also inhibited the autophosphorylation activities of EnvZ and PhoQ from *E. coli*, both with IC_50_s of 1.25 μM. Studies of the inhibitory activity of walkmycin C on the virulence factors of *S. mutans* showed that exposure to walkmycin C at sub-MICs could inhibit biofilm formation, acid tolerance, and competence. Thus, walkmycin C can be used as a potential lead molecule for the development of BHK inhibitors [39].

**Figure 18:**
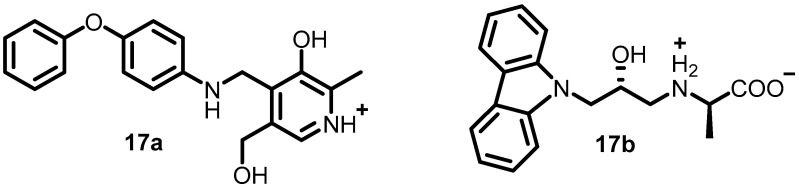
Chemical structures of BHK inhibitors **17a**–**17b**.

In 2010, to identify potent inhibitors of BHK, Henriksen et al. performed virtual screening of a library containing approximately 106 compounds. Forty-nine compounds were found to exhibit potent inhibitory activity, and among them, eighteen compounds were directly evaluated against three different *S. aureus* strains and two *E. coli* strains via disk inhibition assays. Compounds **17a** and **17b** (Figure 18) were the most potent, with G-score values of −7.70 and −7.68 kcal/mol, respectively, and MM-GBSA values of −20.34 and −20.53 kcal/mol, respectively. These compounds can be further optimized for the development of future antibacterial agents [57].

In 2010, Okada et al. screened approximately 1368 cultures of Streptomyces sp. by using differential growth assays and produced different walkmycin derivatives. Among these, walkmycin B (**18**) (Figure 17) had the greatest binding affinity for WalK in *B. subtilis*, with a K_D_ value of 7.63 µM. Furthermore, they measured the autophosphorylation bands densitometrically and calculated the IC_50_ values of **18** against WalK of *S. aureus* (5.7 μM) and *B. subtilis* (1.6 µM) [38].

**Figure 19:**
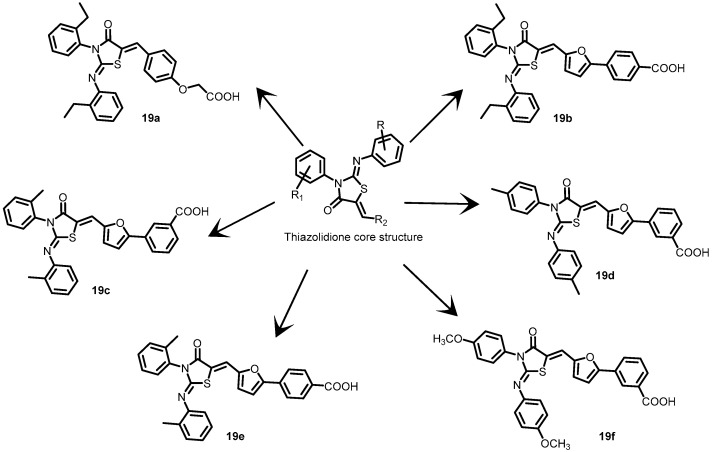
Chemical structures of thiazolidine derivatives **19a**–**19f**.

In another study, Pan et al. (2010) designed and created a series of new 2-arylimino-3-aryl-thiazolidine-4-one compounds based on the core structure of compound **13a** (Figure 14) to develop more potent and less harmful BHK inhibitors. Six derivatives (**19a**–**19f**) (Figure 19) were created by altering the functional groups through cyclization, aldol condensation, substitution, and hydrolysis. The autophosphorylation activity of WalK was inhibited by all six derivatives in a concentration-dependent manner, with IC_50_ values that are comparable to those of **13a** (IC_50_ = 47.9 µM) at 88.35, 61.15, 34.83, 66.68, 22.15, and 82.51 µM [58].

**Figure 20:**
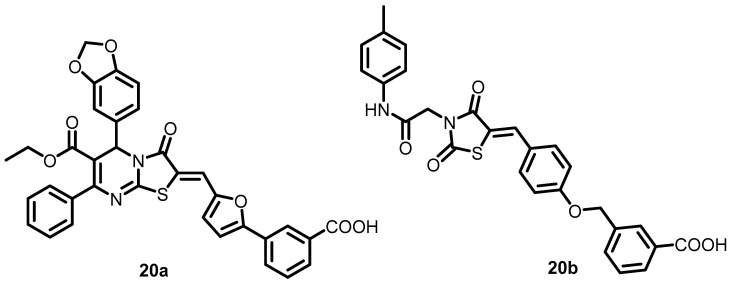
Chemical structures of compounds **20a**–**20b**.

In 2006, Qin et al. initially employed a structure-based virtual screening (SBVS) method to identify potential inhibitors of *S. epidermidis* WalK from a small-molecule library of chemical compounds. Among the 76 candidates that target the WalK ATP binding domain, only seven exhibited significant growth-inhibitory effects on *S. epidermidis*. Compounds **13a** and **20a**–**20b** (Figure 20), which possess a thiazolidione scaffold, exhibited greater ATPase activity of the WalK protein, with IC_50_s ranging from 6.5 to 29 µM. Only the non-biofilm-forming *S. epidermidis* ATCC 12228 was susceptible to **20b**, while **13a** and **20a** were effective against *S. aureus*, *S. pyogenes*, and *S. mutans* [33].

**Figure 21:**
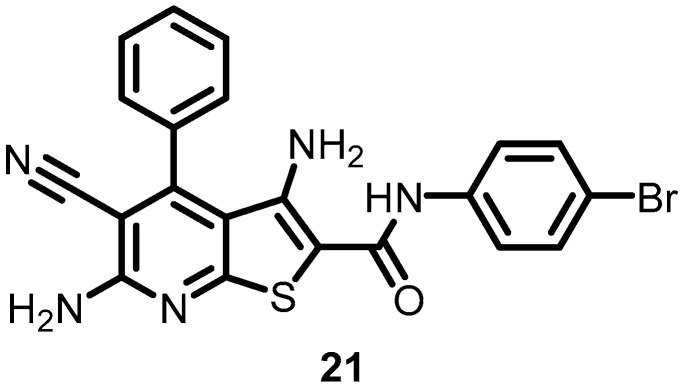
Chemical structure of thienopyridine (**21**).

Gilmour et al. (2005) identified thienopyridine (CAS 332175-01-6) (**21**) (Figure 21) as a novel class of competitive ATP inhibitors of BHKs and analyzed its antibacterial activity by using the HTVS of compound libraries. The results indicated that **21** has a core ring structure that is similar to that of purines, although the exact structural mechanism by which TEP inhibits BHKs is yet unknown. However, its hydrophobic portion may be responsible for cell wall permeation, thereby inhibiting bacterial growth. Competitive ATP inhibition was evaluated using Lineweaver–Burk analysis, and the average Ki value for **21** was found to be 0.62 ± 0.11 µM. Furthermore, **21** could serve as a starting material for novel inhibitors that specifically inhibit BHKs [37].

**Figure 22:**
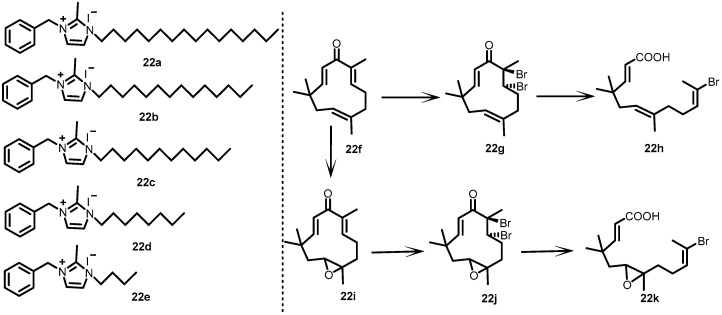
Chemical structures of imidazole derivatives **22a**–**22e** and zerumbone derivatives **22f**–**22k**.

In 2001, Yamamoto et al. developed and examined a series of imidazole (**22a**–**22e**) and zerumbone (**22f**–**22k**) derivatives (Figure 22). Prompted by the inhibitory activity of imidazoles against BHK, the authors screened imidazole derivatives against the autophosphorylation of YycG. Astonishing results were observed for the derivatives, with IC_50_ values ranging from 6.6 to 120 µM. Almost 100 zerumbone derivatives were screened for their ability to inhibit YycG autophosphorylation. However, the results were not positive, and no inhibitor was detected during the study. Then, the authors tried to synthesize zerumbone derivatives by cleaving their cyclic structures. Upon structural modification, the obtained zerumbone derivatives were found to be active (IC_50_: 750–2300 µM). The derivative 29 h was found to be a more potent inhibitor of YycG, with an IC_50_ value of 750 µM [32].

**Figure 23:**
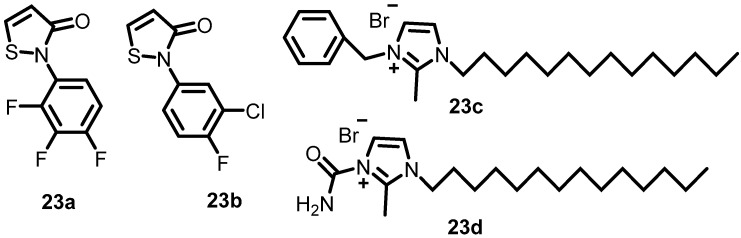
Chemical structures of the thiazole and imidazole derivatives (**23a**–**23d**).

In 1993, Roychoudhury et al. identified compounds that prevent the phosphorylation or dephosphorylation of AlgR2 and the DNA-binding activity of AlgR1, which prevent the production of the alginate gene. In this study, 15 compounds were shown to be effective in screening approximately 25,000 compounds for the inhibition of algD promoter activation. Furthermore, four (**23a**–**23d**) (Figure 23) of fifteen compounds strongly inhibited AlgR1-AlgR2 phosphorylation, AlgR2 kinase activity, AlgR2 phosphatase activity, the DNA-binding activity of AlgR1, and the kinase activities of CheA, NRⅡ, and KinA [41].

## 3. Conclusions

In the present review, we described the roles of the TCS pathway and BHK in bacterial survival and AMR. Furthermore, we also summarize the advancements in the discovery of novel BHK inhibitors in the 21st century. However, despite these massive efforts, none of the discovered inhibitors have entered clinical use or are, to the best of our knowledge, even undergoing clinical studies. To make progress and eventually find new effective antimicrobial medications, challenges must be recognized and overcome. We will use this conclusion section to predict and discuss these possible challenges and obstacles.

Although the chemical structures of the reported inhibitors are diverse, their exact structure–activity relationships cannot be elucidated. Furthermore, progress in the discovery of BHK inhibitors is still in its infancy. Although crystal structures are available, the lack of cocrystals makes it cumbersome to identify potential receptor–ligand interactions. Some studies based on fragment-based and structure-based virtual screening and drug repositioning were implemented to identify BHK inhibitors that exhibited significant inhibitory potential. In the near future, understanding the binding mode and conducting molecular modeling studies will significantly accelerate the discovery of novel BHK inhibitors with greater potency. In addition, researchers can perform more prospective analyses by inducing spontaneous mutants to BHK inhibitors that are under development to elucidate on-target effects and possible resistance mechanisms. Although challenging, designing and developing novel BHK inhibitors is a viable approach for combating AMR.

## Figures and Tables

**Table 1:** BHK and its inhibitors.

Two-Component Systems		Expression System (Bacteria)	Inhibitors	Reference
Histidine Kinase	Response Regulator			
PhoP	PhoQ	*Salmonella typhimurium*	Diaryloxazole	Vo CD, Shebert HL et al., 2017 [30]
			Diarylpyrazoles	
PhoR	PhoB	Gram-negative bacteria	Thiophenes	Velikova N et al., 2016 [31]
			Phenol	
WalK	WalR	Firmicutes	Imidazoliums	Yamamoto et al., 2001 [32]
			Thiazolidiones	Qin Z, Zhang J et al., 2006 [33] Huang RZ et al., 2012 [34] Liu et al., 2014 [35]
			Thiophenes	Boibessot T et al., 2016 [36]
			Thienopyridine	Gilmour R et al., 2005 [37]
			Walkmycin	Okada A et al., 2010 [38] Eguchi Y et al., 2011 [39]
			Signermycin	Watanabe T et al., 2012 [40]
PhoR	PhoP	Firmicutes		
ResE	ResD	Firmicutes	Thiophenes	
EnvZ	OmpR	*Escherichia coli* and relatives	Thienopyridine	
AlgR2	AlgR1	*Pseudomonas aeruginosa*	Isothiazolones	Roychoudhury S et al., 1993 [41]
			Imidazoliums	
VanS	VanR	*E. faecium* (VRE) and *S. aureus* (VRSA)	Thienopyridine	Gilmour R et al., 2005 [37]
KinA	Spo0F	*Bacillus subtilis* and relatives		
			6-oxa isosteres	Kanojia RM et al., 1999 [42]
			Indoles	Weidner-Wells MA et al., 2001 [43]
			Benzimidazoles	Weidner-Wells MA et al., 2001 [43]
			Benzoxazoles	Weidner-Wells MA et al., 2001 [43]
CheA	CheY	Motile bacteria of all phyla		Welch M et al., 1998 [44]
NtrB or NRI	NtrC or NRII	*Escherichia coli* and relatives	Diaryltriazoles	Pioszak A A et al., 2003 [45] Kanojia RM et al., 1999 [42]
			6-oxa isosteres	
HK853	RR468	*Thermotoga maritima*	Adenines	Wilke KE et al.,2015 [46] Goswami M et al.,2018 [47]
			Benzothiazoles	Wilke KE et al.,2015 [46]
CckA	CckA	*Caulobacter crescentus* and relatives	Diarylpyrazoles	Vo CD et al.,2017 [30]

## Data Availability

Not applicable.
